# GAFchromic EBT film lateral resolution and contrast reproduction in the UV-blue range

**DOI:** 10.1038/s41598-024-78100-3

**Published:** 2024-11-22

**Authors:** Francesco Garzella, Giacomo Insero, Antonella Battisti, Antonella Sgarbossa, Tommaso Mello, Franco Fusi, Giovanni Romano

**Affiliations:** 1 Department of Experimental and Clinical Biomedical Sciences “Mario Serio”, University of Florence, Florence, Italy; 2 Department of Surgical, Medical and Molecular Pathology, and Critical Care Medicine, University of Pisa, Pisa, Italy; 3 NEST, National Research Council - Nanoscience Institute (CNR-NANO) and Scuola Normale Superiore (SNS), Pisa, Italy

**Keywords:** Optics and photonics, Applied optics, Optical materials and structures, Optical techniques, Materials science, Materials for optics, Techniques and instrumentation

## Abstract

The sensitivity of radiochromic films to UV-blue light is increasingly considered for light dosimetry purposes, owing to their bidimensional detection capabilities and ease of use. While film response to radiation intensity has been widely investigated by commercial scanners, spatial resolution studies remain scarce, especially for small field-of-view applications. These are of growing interest due to the antimicrobial or photo-bio-stimulating effects of UV-blue light sources in in vitro, ex vivo and in vivo models, where precise knowledge of irradiation conditions with adequate spatial resolution is crucial. In this study, we report the spatial lateral resolution and contrast reproduction of GAFchromic EBT2 and EBT3 models. Upon film irradiation by a 405 nm laser source or 365 nm LED, a confocal microscope setup was employed to read the film response at 405, 470, 488, 532 and 570 nm wavelengths, with radiant exposure of 10–70 J/cm^2^. The measured lateral resolution ranged from 8 to 33 μm. The film capability to reproduce contrast across various spatial frequencies (4–14 lines/mm) was evaluated using modulation transfer function analysis with irradiation performed at 365 nm and 405 nm, revealing a pronounced dependency on both radiant exposure and reading wavelength. These results confirm the film capacity to detect and resolve light intensity variability with a ~ 10 μm resolution, with notable applications in micro-beam profiling and light dosimetry.

## Introduction

Radiochromic films, and in particular GAFchromic films (GCFs), are a family of self-developing materials, made by an active layer containing a marker dye that undergoes a polymerisation process as a result of the interaction with ionising radiation, both electromagnetic and particle^1^. The formation of polymeric chains is accompanied by a change in the optical absorption properties^2^ that is reflected by a macroscopic film’s colour change. Besides, polarisation processes can be also present in the case polarised light is used to measure GCF optical density in the post-irradiation phase^3,4^.

Along the 19th century, GCFs have established as one of the possible standards for gamma- and X ray dosimetry^5–7^, thanks to their availability and sensitivity to a wide range of wavelengths up to the visible domain, besides their inherent capacity for 2D detection. Nowadays the main GCFs applications include absolute^8^ X-ray/gamma-ray dosimetry^9–12^, with rising applications in small field dosimetry^13^ and microbeam profiling^14,15^, where their portability, accuracy, resolution and bi-dimensional response ability play a key role^16,17^. In the last years, due to their easiness of use and 2D detection capabilities, GCFs are being increasingly considered for UV and, partly, visible dosimetry^18–21^. In particular, both EBT2 and EBT3 models show a response to UVA, UVB and UVC radiation^22–24^ with main applications to the study of sun-related UV exposure and its associated health issues^25–28^. More recently, GCF are gaining consideration for beam profiling and as a two-dimensional dosimeter with micrometer spatial resolution. Yusof et al.^29^ used GAFchromic EBT3 to investigate the properties of UV radiation via UV light emitting diodes (LEDs) with two different peak emission wavelengths (365 and 375 nm). Their results indicate the effectiveness of EBT3 film for quantitative measurements of UV exposure.

Despite their potential for beam profiling, GAFchromic film applications are limited by the resolution of systems currently used to analyse their response, such as flatbed scanners or densitometers. Be it a medicine-, microscopy- or technology related application, GCFs response to UV radiation is best exploited when coupled with suitable optical reading and image-analysis techniques. In this regard, the literature lacks studies that establish the spatial resolution of GCF, a crucial parameter for accurately interpreting imaging data. Notable applications are in the field of high-resolution beam profiling, small field dosimetry and the use of UV-blue light in antimicrobial phototherapy and regenerative medicine, where *in vitro* samples are irradiated and analysed by optical microscopy setups and techniques^30,31^. Currently there are a few methods for evaluating the spatial resolution of GCFs. Myiatake et al.^32^ recently developed an approach based on the modulated transfer function (MTF) to analyse the GAFchromic HD-V2 model by employing a line space pattern and evaluating it using either a flatbed scanner and a test bench microdensitometer (TMBD). Both approaches were constrained by the spatial lateral resolution of detector of 83.3 μm for the flatbed scanner and 13 μm for TMBD. In this work, we employed the high spatial resolution of a confocal microscope to measure the MTF, that represents the correlation between spatial frequency and contrast, and to accurately evaluate the optical performances of GCF. The space lateral resolution of GCFs when irradiated in both the UV and blue regions is strictly connected with the polymerisation processes occurring within the active layer and underlying the film darkening^2,33^, being ultimately limited by their occurrence also in nearby non-irradiated film regions. Unfortunately, most GCF models, including EBT3, integrate a protective layer that prevents part of the UV and visible wavelengths to reach the active layer, thus reducing the film overall performance in those spectral regions. This does not prevent GCFs to be considered as a useful detection tool in the UV-blue region, provided it is properly characterised in terms of spatial resolution and contrast reproduction.

In this work we report on the characterisation of GAFchromic EBT2 and EBT3 lateral spatial resolution via confocal microscopy when irradiated at 365 or 405 nm and read with wavelengths in the range 405–670 nm; besides, the influence of 405 nm continuous/pulsed irradiation conditions on the film’s response was enquired. The approach is based on two sequential steps: film irradiation (“writing”) and subsequent reading. In the first step, a light source is used to irradiate (darken) the film via the photo-polymerisation process, while in the second reading step the film’s response is measured in terms of transmitted light variations (spectral optical density). The film resolution was evaluated via direct measurement of its response to a diffraction-limited single point excitation (irradiation phase) and reading with visible wavelengths using a confocal microscope setup. In the UV range (365 nm) the film resolution was measured via the Edge Spread Function (ESF) onto darkened film, determining the maximum lateral resolution achievable. Lastly, the film capacity to reproduce contrast at different spatial frequencies (MTF) was evaluated by reading its response with a confocal microscopy setup.

## Materials and methods

### Sample preparation

**Fig. 1:**
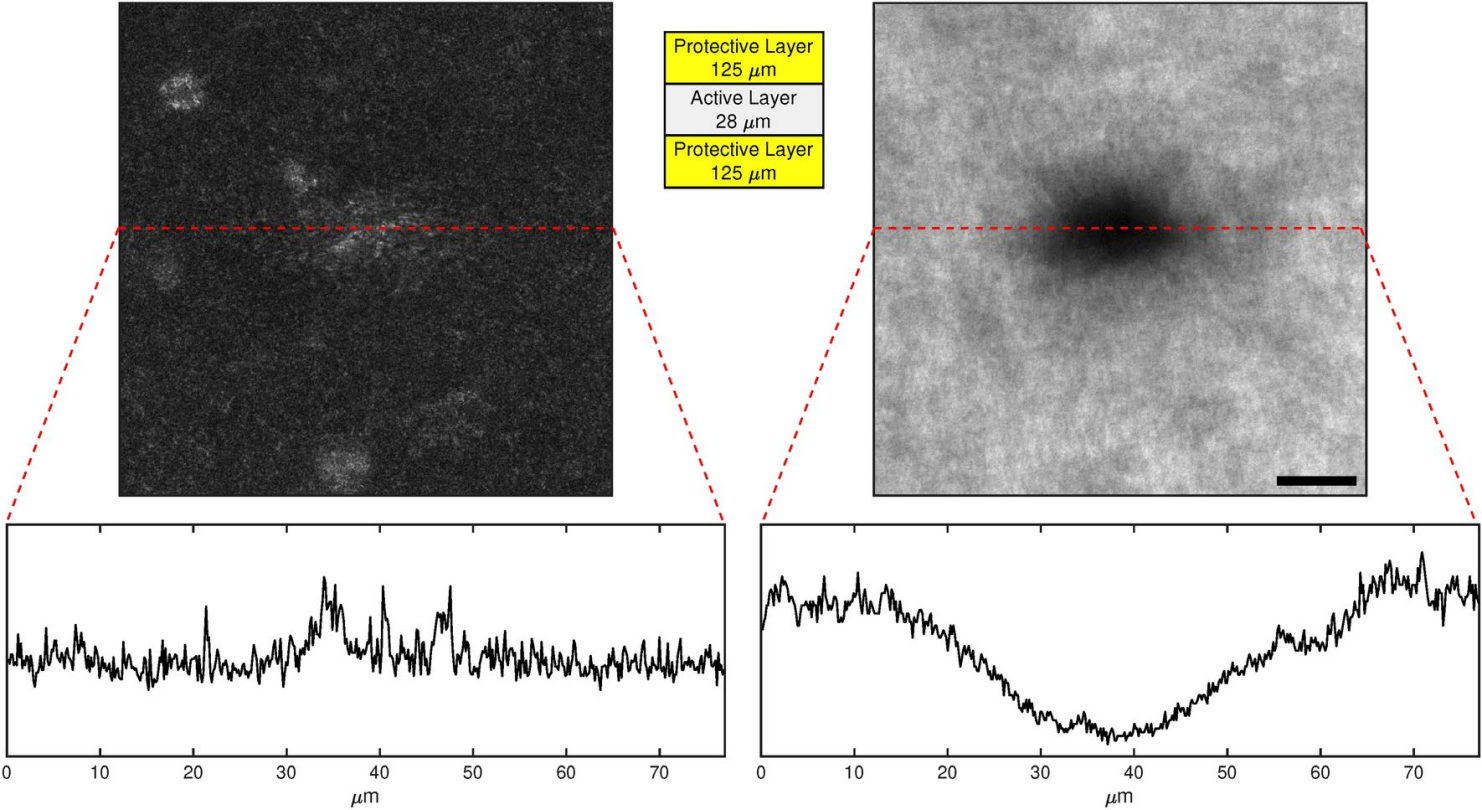
Comparison of two reading modes following GCF EBT3 film irradiation at 405 nm: reflected (left) and transmitted (right) light mode, both acquired at 488 nm. The bottom part reports the profile along the red line. Scale-bar is 15 um. In addition, the structure of GAFchromic EBT3 film is also reported at the centre of the figure. The film is comprised of an active layer, nominally 28 μm thick, sandwiched between two 125 μm matte-polyester protective layers.

The self-developing films used for the experiments are the GAFchromic EBT2 and EBT3 models (Ashland Advanced Materials, Bridgewater, NJ, USA). The EBT3 film is composed by an active layer, nominally 28 μm thick, sandwiched between two 125 μm matte-polyester substrates of Mylar (see Fig. 1), while the EBT2 film has an asymmetrical structure^34^ composed of a polyester layer (50 μm ), adhesive layer (20 μm), active layer (28 μm), polyester layer (175 μm). The active layer contains the light-responsive monomer (a poly-crystalline and substituted diacetylene), stabilisers and other components^34^. A virgin A4 film as from the factory was cut into 1 cm × 1 cm samples; ambient light exposure was minimised throughout the whole experimental procedures. Samples were then sandwiched with a drop of immersion oil (Leica Immersion Oil, NA = 1.5180, Leica Microsystems GmbH, Mannheim, Germany) between a glass slide (CNWTCMed, Chongqing, China) and a cover-slip (#1.5, 0.170 mm, VWR, Germany). This sample preparation is common to all the experiments presented in this work. As mentioned, the film is treated to be poorly sensitive to visible light, reducing the available spectra to the UV/blue region. The last available GCF EBT3 model protective layer is more efficient than EBT2 one to protect the active layer from visible light.

### Experimental layout for film irradiation

Prior to concentrate on GCF irradiation with 405 nm, preliminary experiments were performed with both EBT2 and EBT3 models where the following irradiation wavelengths were considered in the visible range: 405, 470, 488, 532 and 670 nm. These measurements are intended to show how the film response is strongly depressed for wavelengths longer than 405 nm, which is why the lateral spatial resolution measurements were focused on the two cases of irradiation at 405 nm and 365 nm only. Film irradiation with 405 nm sources and subsequent film reading experiments were performed using two inverted confocal microscope setups: one for continuos wave (CW) film irradiation, the other for pulsed film irradiation. In both cases, a laser beam is tightly focused onto a GCF portion, thanks to a high numerical aperture objective. The same respective microscopy setup was then used to read the film, in different conditions. To choose the best optical configuration for film reading between the transmitted and reflected light mode, preliminary measurements were performed on both EBT2 and EBT3 models. Both films were irradiated with a 405 nm laser source via the “bleaching point mode” of the confocal microscope setup. The resulting polymerised spot was subsequently read to analyse image contrast in both modes. The irradiation spot size was defined as the diameter $d_{s}$ of the Airy disc (Rayleigh criterion), following the relation $d_{s}=1.22\frac{\lambda }{NA}$, while the light power used were 50, 51, 58 and 150 μW respectively for 405 nm, 488 nm, 532 nm, and 670 nm wavelengths.

*405 nm CW irradiation*: A Leica SP8 STED 3X confocal microscope and accessories (lasers, detectors and objectives) was used (Leica Microsystems GmbH, Mannheim, Germany), equipped with two light sources emitting at 405 nm and in the range 470–670 nm, two alkaline Photomultipliers (PMTs), one Leica Hybrid Detector (HyD), a transmitted light detector and the following objectives: 40× (NA = 1.3), 63× (NA = 1.4), 100× (NA = 1.4). The violet CW light (405 nm) is provided by the built-in diode laser while the built-in Super Continuous Laser (or White Light Laser, WLL) combined with an Acousto-Optic Beam Splitter (ABOS) provides all the laser lines in the range from 470 nm to 670 nm. The WLL master percentage and the diode power percentage are kept constant (85% for both) among all the experiments. The film irradiation was performed with the 405 nm CW laser via the “bleaching point mode” of the confocal microscope setup, while the reading was performed through selected WLL lines, by recording the transmitted light image, at the following reading wavelengths: 405, 488, 532 and 670 nm; the scan speed was set to 600 Hz (lines per second). Laser power was measured by a power meter (Nova OPHIR, Ophir Spiricon Europe GmbH Ophir Distribution and Calibration Center, Darmstadt, Germany) at the objective’s focus level. During measurements, the microscope was set on “bleach point” mode from the built-in Leica software (LAS X, Leica Microsystems GmbH, Mannheim, Germany). The output power of the single lines of the WLL was measured adjusting the percentage through the AOBS keeping the master power at 85%. Images acquired with the SP8 confocal setup have a size of 512 × 512 pixels for a Region Of Interest (ROI) of 76.90 μm × 76.90 μm or with a size of 3840x3840 pixels for a ROI of 246 μm × 246 μm. For both configurations, pixel size was 0.15 μm.

*405 nm pulsed irradiation*: A Leica TCS SP5 inverted confocal microscope was used (Leica Microsystems AG, Wetzlar, Germany), equipped with an external pulsed diode laser at 405 nm and 470 nm (irradiation mode, Picoquant Sepia Multichannel Picosecond Diode Laser, PDL 808), an Ar laser line for excitation at 488 (both irradiation and reading mode) and 514 nm (irradiation mode) and a transmission PMT for detection. Laser repetition rate was set to 40 MHz, unless otherwise stated. Pulsed laser was used with no power attenuation. Image size was 512x512 pixels, for a ROI of 155 μm × 155 μm and a pixel size of 0.303 μm, and scan speed was set to 400 Hz (lines per second). The objective was a HCX PL FLUOTAR 100× 1.30NA oil immersion objective (Leica Microsystems) and the pinhole aperture was set to 1 Airy (163.3 μm confocal aperture). Film’s irradiation was performed either with the 405 nm and the 470 nm pulsed laser while the reading was done through the 488 nm CW laser. The pulsed laser power was measured by a power meter (843-r/818-UV, NewPort, Irvine, California). During these measurements, the microscope was set on “bleach point” mode from built-in Leica software (LAS, Leica Microsystems GmbH, Mannheim, Germany).

*365 nm irradiation*: Ultraviolet film irradiation was performed by considering an external LED source (precisExcite-2, CoolLED, USA) emitting CW radiation peaked at 365 nm (FWHM = 15 nm), as UV wavelengths were not available on our confocal microscope systems. The LED source was coupled with an optical fibre, providing a total radiant power of 53 mW (Nova OPHIR) from the fibre exit. Post-irradiation film reading was performed by a confocal setup (SP8), as described below.

### Layout to measure spatial resolution

The lateral spatial resolution of GCF-EBTs and its dependence on the radiant exposure was investigated in the two cases of 405 (CW and pulsed) and 365 nm irradiation, corresponding to the use of a laser line provided by the confocal setup and an external LED source, respectively. This necessarily implies the adoption of two different approaches to evaluate the GCF resolution: in the 405 nm irradiation case, the lateral resolution was investigated by imaging the film response to a point-like illumination; in the 365 nm writing case, the resolution was derived by acquiring the film response when irradiated through a specific test chart containing a pattern that produces sharp edges on the exposed film.

### Resolution at 405 nm

To characterise GCF EBTs lateral resolution, the film was irradiated at 405 nm (both CW and pulsed) in the single bleach-point mode. The produced spot was then read at different reading wavelengths, corresponding to 405 nm, 488 nm, 532 nm, and 670 nm for the CW setup and 488 nm only for the pulsed setup. The corresponding spot image was analysed in terms of line profile along the two axes of the ellipse-shaped spot produced. The lateral resolution was defined in terms of the Full Width at Half Maximum (FWHM) calculated by a two-dimensional Gaussian function (Eq. 1) used to fit the spot profile, in the hypothesis that in the chosen experimental conditions the spot corresponds to the Point Spread Function (PSF) of the film/microscope reading system. As the obtained spots have an elliptic shape, the lateral resolution was separately defined in relation to the minor (c) and major (b) axes, respectively. Equation (1) is valid for x-y coordinates corresponding to the major and minor axis of the elliptical spot shape, respectively, being the FWHM defined as $2\sqrt{2ln2}\sigma$ and being $\sigma _x$ and $\sigma _y$ the standard deviation along the *x* and *y* axis, respectively,

1 $$\begin{aligned} f(x, y)=A\cdot \exp {\left( -\frac{(x-x_0)^2}{2\sigma _x^2}-\frac{(y-y_0)^2}{2\sigma _y^2} \right) }+d \end{aligned}$$

For 405 nm cw irradiation, each point is obtained by a 4 s irradiation at 50 μW for a total energy of 0.2 mJ per point. For the pulsed 405 nm laser source, each point is acquired every 30 s with a measured power of 10 μW and a total energy of 0.3 mJ.

All the resolution results obtained with the bleach point method have an associated relative uncertainty which, for every reading wavelength, was evaluated as 3–9% with larger values as radiant exposure increases. These relative uncertainties were derived as the relative mean squared deviation across ten repeated measurements.

To provide a more visual description of the film resolution, we were inspired by the resolution limit defined by Rayleigh’s criterion in its original formulation, based on the human visual system and can provide sufficient contrast for an observer to distinguish two separate objects within an image. For this purpose, we considered the exemplary case of 405 nm CW irradiation of the GCF, where several pairs of points were irradiated in the bleaching point mode with decreasing set distances and read at 488, 532 and 670 nm (as described in the Results and Discussion section). For each couple of points we have extracted the corresponding line intensity profile passing through the point centroids. Each profile was fitted with two Gaussian functions of the same width and centred in $x_1$ and $x_2$ respectively, added to a linear background. Then, we defined a parameter *R*(*d*) as the ratio between the signal calculated in the intermediate position between the two peaks, *i.e.* $(x_1+x_2)/2$, and the minimum value between the two peak amplitudes, while $d=|x_1-x_2|$ represents the distance between the two Gauss peak positions. As a condition of Rayleigh’s criterion, we defined the resolution to be equal to the distance between the two peak *d* for $R\equiv 2$, meaning that the signal calculated in the intermediate position is half the average between the two peak amplitudes. The uncertainty has been obtained by proper error propagation of the fit parameters (see Supplementary Notes).

### Resolution at 365 nm

**Fig. 2:**
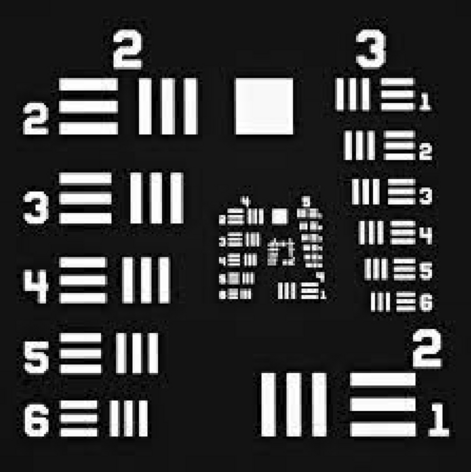
Schematic representation of negative USAF-1951 target showing the appearance of groups 2 and 3 and high order groups.

The film resolution for 365 nm-induced darkening was measured via evaluation of the Edge Spread Function (ESF), which represents a sensor optical response to an object sharp edge and whose first derivative is the Line Spread Function (LSF), which represents the integral of the Point Spread Function (PSF) on one direction. The LSF’s FWHM represents then the resolution along an axis. The measurement scheme is then: (i) obtain a well-definite darkened portion of the film and measure the edge line profile = ESF; (ii) derive the ESF to obtain the LSF; (iii) obtain the resolution by measuring the FWHM of the LSF. This approach can be applied to determine the GCF resolution when a point-like irradiation is not available^35^. To perform these measurements, a USAF-1951 resolution test chart was used, originally defined by the U.S. Air Force (MIL-STD-150A standard of 1951) and formed by a series of specimens with precise spatial frequency and squares, producing sharp edges on the exposed film. A schematic representation of a USAF-1951 target is reported in Fig. 2 (for a more detailed representation of how the pattern is transferred to the film see Supplementary Figure 1). The test chart was placed in perfect contact with a film portion to prevent shadow formation and shined by the 365 nm LED source coupled with the optical fibre. The fibre end was placed above the test chart and orthogonal to it, at a distance of a few centimetres. In these conditions, the UV spot at the chart level has a constant irradiance of about $( 10.3 \pm 0.1)~\mathrm {mW/cm^2}$, as measured by a power meter (Nova OPHIR, Ophir Spiricon Europe GmbH Ophir Distribution and Calibration Center, Darmstadt, Germany). The edges of the chart shapes where imaged via a confocal microscope using the SP8 setup with a size of 3840 × 3840 pixels for a ROI of 246 μm × 246 μm and a reading wavelength of 488 nm. According to Kohm et al.^36^ for each data set, sample was imaged to have the edge aligned either with one axis or the other. The obtained edge profile in terms of pixel value vs point position was averaged along the transversal edge direction obtaining the ESF and then derived through the finite difference method, obtaining the LSF which was fit by the following equation:

2 $$\begin{aligned} f(x)=[(a-d)\cdot e^{-(\frac{x-b}{c})^2}+d]H(b-x)+a\cdot e^{-(\frac{x-b}{c})^2}H(x-b) \end{aligned}$$

Where *a*, *b* and *c* are respectively the amplitude, the centre and the standard deviation of the Gaussian function, *d* is an offset and *H* is the Heaviside step function. The FWHM is then expressed as $2\sigma \sqrt{2ln2}$.

### Layout to measure film contrast reproduction

The film capacity to reproduce contrast as a function of the space frequency was measured by considering the modulation transfer function (MTF), representing the capability of a sensor to transfer the contrast from the object to the image plane^37^. The general approach to measure the MTF considers a series of specimens exhibiting precise increasing spatial frequency $\nu$ expressed in line pairs per mm (lp/mm); the targets are imaged by the sensor under test and the corresponding contrast is measured. The plot of the ratio of image contrast over target contrast against $\nu$ represents the MTF. This was performed for both the EBT2 and EBT3 models by illuminating the USAF-1951 test chart, which contains patterns at increasing frequency. Due to the necessity of illuminating the test chart, no confocal illumination could be used. To reproduce the same irradiation wavelengths considered for the resolution measurements, the following two sources were used to uniformly illuminate the whole chart: (i) the 365 nm-peaked LED previously cited; (ii) an additional 400 nm-peaked LED source (precisExcite, CoolLED, USA). Irradiated films were imaged by the Leica SP8 confocal setup (the confocal images of the USAF-1951 different patterns on GCF EBT3 are reported in Supplementary Figure 2). The resulting image pattern reproduced onto the GCF film was acquired and the contrast *C* was defined as reported in Eq. (3):

3 $$\begin{aligned} C(\nu )=\frac{I_{max}(\nu )-I_{min}(\nu )}{I_{max}(\nu )+I_{min}(\nu )} \end{aligned}$$

where $I_{max}$ and $I_{min}$ are defined as the maximum and minimum values of the imaged target pattern (expressed for us in pixel values), at a given frequency (see also Supplementary Figure 3); *C* = 1 corresponds to a perfect (maximum) contrast while *C* = 0 corresponds to no contrast (a uniformly grey target image). The general definition of MTF is shown by Eq. (4):

4 $$\begin{aligned} MTF(\nu )=\frac{C_{image}(\nu )}{C_{target}(\nu )} \end{aligned}$$

where $C_{image}$ corresponds to $C(\nu )$ defined by Eq. (3) and $C_{target}$ is the target (USAF-1951 test metal chart) contrast which, in our case, was assumed to be equal to unity due the construction features of the chart itself. The USAF target has two crucial parameters defining the resolution: the element number and the group number. The scales and dimensions of the bars are given by the Eq. (5):

5 $$\begin{aligned} res(\mathrm {lp/mm})=2^{group+(element-1)/6} \end{aligned}$$

In Table 1 the values of the resolution are reported for the six elements of both groups two and three.

**Table 1:** This table reports spatial frequency values for the six elements of target USAF-1951 groups two and three.

Group	1	2	3	4	5	6
2	4	4.49	5.04	5.66	6.35	7.13
3	8	8.98	10.08	11.31	12.70	14.25

Frequency is expressed in lp/mm.

During film irradiation, the fibre was kept vertical over the sample, producing a light spot whose irradiance inhomogeneities (at the sample level) were estimated of about 10%, as measured by the power meter. The 365 nm irradiance was 10 μW/cm^2^ and 70 μW/cm^2^ for 405 nm. Supplementary Figure 2 Images of USAF-1951 target for MTF measurements were acquired in the light transmission mode of the CW setup using lower magnification objective (40× objective, 1024 × 0124 pixels, 387.6 μm × 387.6 μm ROI) and a reading wavelength of 488 nm.

### Layout to measure the field of View

To test one possible application in the optical microscopy field, an objective Field of View was measured. The Field of View (FoV) is the extent of the observable area through the objective lens. The FoV was first calculated through the formula $FoV=FN/M_{O}M_{eye}$ where *FN* is the field number and $M_{O}$ and $M_{eye}$ are respectively the objective and the eyepiece magnification. Then, FoV direct measurement was obtained by shining the film with the DAPI line of the built-in lamp of SP8 using 100x objective. The lamp has a broad emission peaked at 360 nm. The produced dark spot onto the GCF was read using a lower magnification objective (40×, NA = 1.3 objective; 5080x5080 pixels and 387.6 μm × 387.6 μm) at 488 nm, a line profile across its diameter was measured, and its gradient was fit with a double Gaussian function. The Gaussian centre positions allowed to estimate the FoV dimension.

### Image and data analysis

All images were provided by Leica Software as proprietary format (.lif) in Gray scale 8-bit (SP8-CW) or 16-bit (SP5-Pulsed). All images were processed using the Fiji free software^38^ and exported as lossless format (.tiff). Exported images are then processed on Matlab (The MathWorks, Inc., USA), where 16-bit images are converted to 8-bit images and analysed by custom scripts.

## Results and discussion

Table 2 resumes the different results obtained about GCF characterization in terms of lateral spatial resolution, contrast reproduction and use as a tool to measure an objective’s field of view.

**Table 2:** Measurement parameters and outcomes.

Measurement type	Irradiation parameters	Reading parameters (nm)	Outcome
preliminary irradiation tests (visible range)	$\lambda$ = 405, 470, 488, 532, 670 nm	$\lambda$ = 488	405 nm as the chosen visible irradiation $\lambda$
EBT2 and EBT3 film lateral spatial resolution	CW 405 nm laser	$\lambda$ = 405, 470, 488, 532, 670	Resolution @405nm (FWHM)
	pulsed 405 nm laser	$\lambda = 488$	Resolution 405nm (FWHM)
	CW 405 nm laser	$\lambda$ = 488, 532, 670	Resolution @405nm (Rayleigh’s Criterion)
	365 nm LED	$\lambda = 488$	Resolution @365nm from film MTF measurement
EBT2 and EBT3 film contrast reproduction capability	365, 400 nm LEDs USAF-1951 chart	$\lambda = 488$	Film MTF
Objective field of view	360 nm laser (DAPI line) 100× objective	$\lambda = 488$	100x FoV @360nm

Figure 1 reports the different appearances of the EBT3 film darkened region in both modes, together with an exemplary line profile of the image grey levels along one of the spot symmetry axis. A higher contrast was obtained in the transmission mode for all the chosen irradiation and reading wavelengths. For this reason, the confocal microscope “bleaching point” and “transmission reading” modes were chosen for GCF writing and reading, respectively. It should be noted that the method used for estimating the lateral resolution is independent of the polarisation of the light used during the reading phase, as polarisation only influences the parameter *A*^3,4^ and not the width parameter $\sigma _x$ or $\sigma _y$. All the more so if using non-polarised light in the reading phase.

### Lateral spatial resolution

**Fig. 3:**
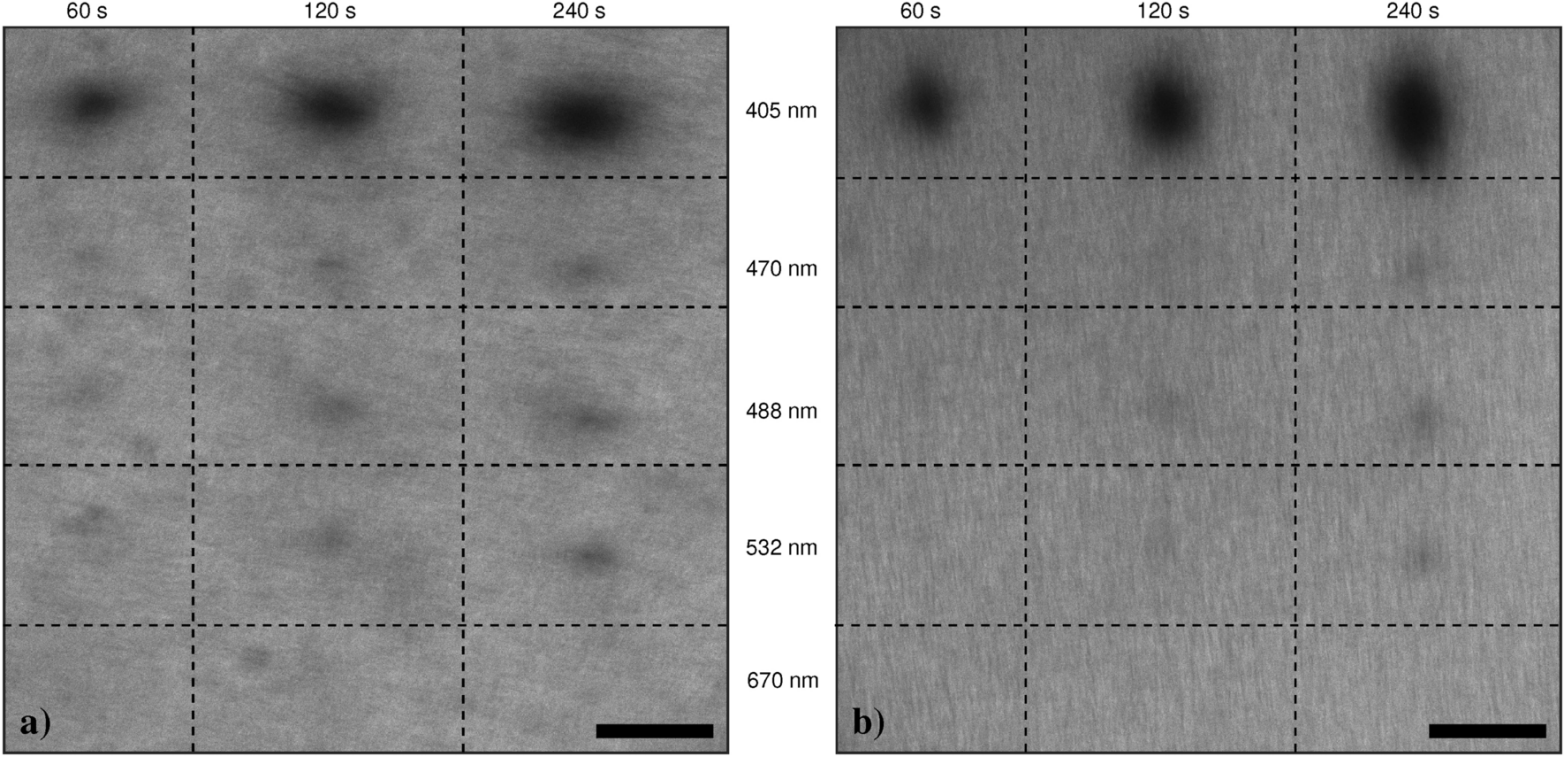
EBT3 (**a**) and EBT2 (**b**) film darkening at different irradiation wavelengths: 405 nm, 470 nm, 488 nm, 532 nm and 670 nm, from top to bottom. For both films 405 nm produces a strong effect while the effects of the others four wavelengths decreases with the increasing of the wavelength. Image reading is performed at 488 nm. Scale-bar is 40 μm.

Figure 3 shows GCF EBT3 (a) and EBT2 (b) darkening obtained with single spot irradiation mode (bleaching point) at 405, 470, 488, 532 and 670 nm (Leica SP5 and SP8 confocal setup lines) for a time of 60s, 120s and 240s. For both films, a dependence of the response upon radiant exposure is clearly visible only in the case of 405 nm irradiation, while the 532 nm and the 670 nm lines are completely ineffective. Minor effect can be measured for 470 nm and 488 nm. It is worth noticing that the radiant exposure is also referred to as *dose* in the phototherapy field, *i.e.* applications related to the use of therapeutic non-ionising radiation in the UV, visible and near infrared range. Of course, the use of this term has to be properly acknowledged not to be confounded with its correct use (in the ionising radiation field) associated with the Gy measurement unit in the case of absorbed dose. Table 3 resumes the light power, spot dimensions as a function of the irradiation wavelength. As expected, starting from a visual inspection it is clear that irradiation at 405 nm corresponds to the most efficient film darkening; in view of dosimetry and beam profiling applications, 405 nm is the only visible wavelength that will be considered in this work for the film writing phase. As shown in Supplementary Figure 4, removal by peeling of the protective layer left the whole film sensitive to visible light. Notably, the darkening occurs in an anisotropic way so that the spot is elliptical. This evidence may be explained by the presence of a well-visible weft (see also Supplementary Figure 5), presumably due to the fabrication process used by the producer.

**Table 3:** Parameters for preliminary irradiation tests in the visible range, for both GCF EBT2 and EBT3 models.

Wavelength (nm)	Spot size (nm)	Power (μW)
405	353	50
470	410	51
488	425	51
532	464	58
670	584	150

### Resolution for 405 nm irradiation

**Fig. 4:**
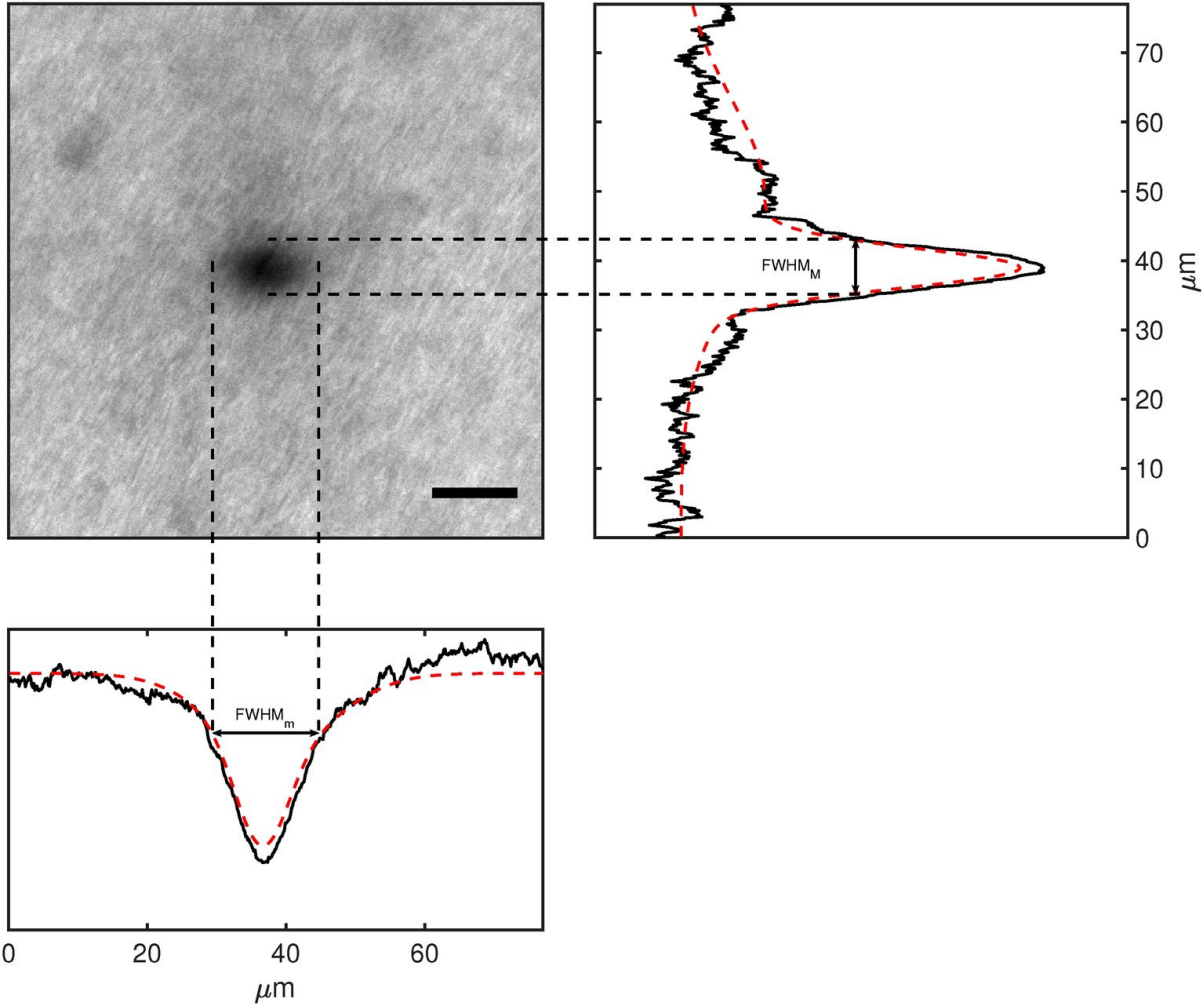
Film darkening resulting from 405 nm writing (left) and the corresponding line profile on major (right) and minor (bottom) axes with an indication of FWHM parameter, estimated via Gaussian fittings (red dashed curves). $\hbox {FWHM}_\textrm{m}$ is 23.8 μm and $\hbox {FWHM}_\textrm{M}$ is 36.7 μm. Scale-bar is 15 μm.

**Fig. 5:**
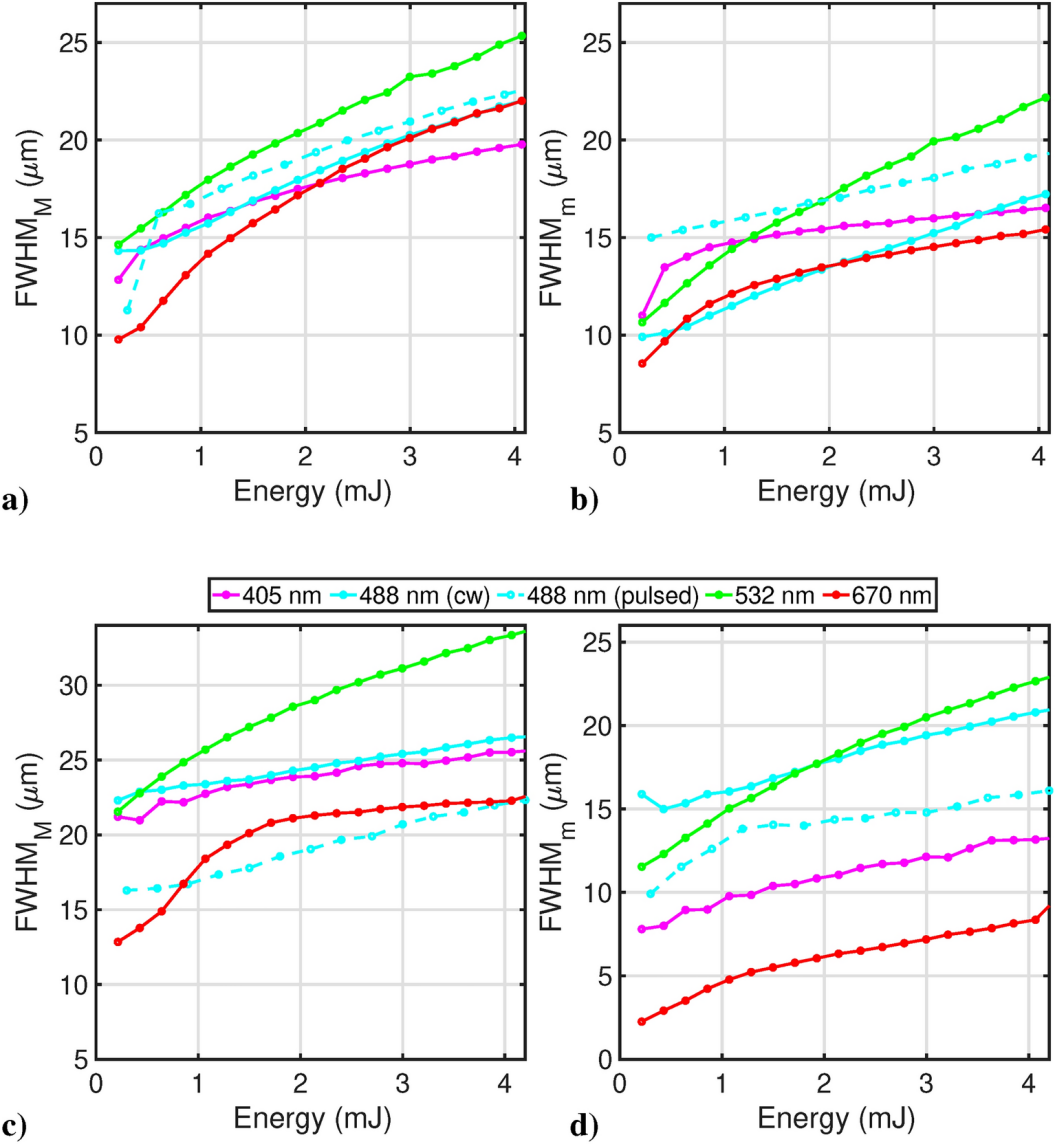
Film resolution, in terms of FWHM, following both CW and pulsed 405 nm irradiation, along the minor ($\hbox {FWHM}_\textrm{m}$) and major ($\hbox {FWHM}_\textrm{M}$) axis respectively for GCF EBT2 (**a** and **b**) and GCF EBT3 (**c** and **d**); reading is at 405 nm (violet), 488 nm (cyan solid for CW and cyan dashed for pulsed 405 nm writing), 532 nm (green) and 670 nm (red).

Figure 4 shows the film darkening process upon irradiation with the 405 nm CW source in the bleach point mode. The elliptic shape for the produced spot indicated that darkening occurs in a non-isotropic way, which justifies the use of a two-dimensional Gaussian profile for spot analysis. Figure 5 reports the comparison between the lateral resolution on the minor and major axis of this ellipse-like shape, respectively, for both GCF EBT2 (a and b) and GCF EBT3 (c and d) at the various reading wavelengths and for irradiation with both cw and pulsed 405 nm sources. The darkening effect of all the reading wavelengths is negligible with respect to the initial 405 nm irradiation phase due to the limited dwell time.

Table 4 reports the resolution values at 4.1 mJ irradiation energy. The spot FWHM increases at increasing irradiation energy. This could be explained by the presence of scattering phenomena, associated with photo-induced polymerisation in regions out of the illumination spot; at the same time, the mechanisms co-opting monomers to form long polymer chains can be also recalled to explain why the dark region is much bigger than the illumination spot. At increasing intensity, the contrast increases as expected, being associated to a higher polymer density. A strong dependence of the spot FWHM on the reading wavelength is also evident, which can be explained by the presence of characteristic peaks in GCF absorption and scattering properties, which was already ascertained by previous work on a different GCF model containing the same class of monomers^19,39^: the resulting polymer shows a much higher absorption in the green region resulting in a more transparent blue/red part of the visible spectrum. As a confirmation, Supplementary Figure 6 reports the comparison of the final point of Fig. 5, dashed blue line, and another bleached point obtained by irradiating continuously for the same amount of time; the appearance is comparable.

**Table 4:** This table reports FWHM values along the minor ($\hbox {FWHM}_\textrm{m}$) and major ($\hbox {FWHM}_\textrm{M}$) axes for both EBT3 and EBT2 films for all the wavelengths showed in Fig. 5. All values are obtained for a total energy of 4.1 mJ.

	FWHM (μm, EBT3 model)		FWHM (μm, EBT2 model)	
Reading wavelength (nm)	$\hbox {FWHM}_\textrm{m}$–$\hbox {FWHM}_\textrm{M}$		$\hbox {FWHM}_\textrm{m}$–$\hbox {FWHM}_\textrm{M}$	
405	13–25		16–19	
488 (CW)	20–26		17–22	
488 (PULSED)	16–22		19–22	
532	22–33		22–25	
670	8–22		19–22	

**Fig. 6:**
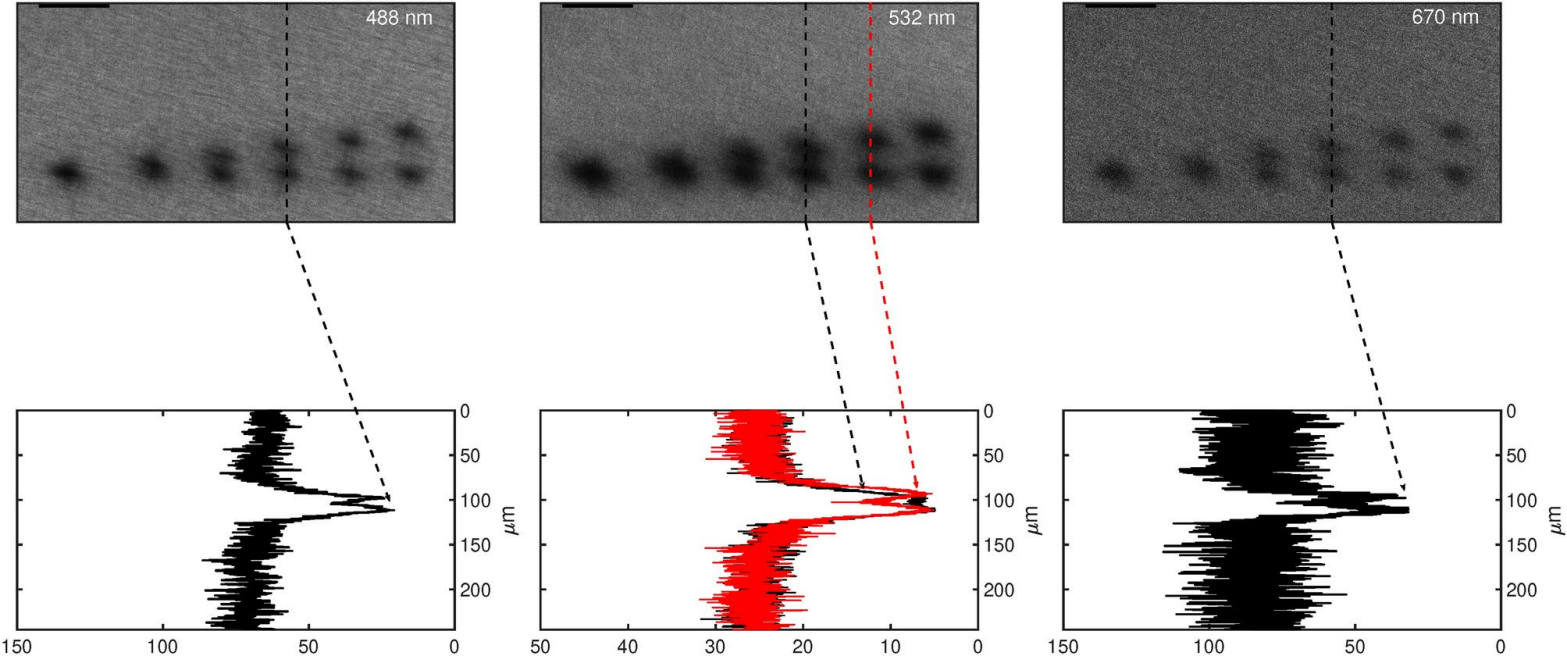
Pairs of bleaching points with decreasing distance and corresponding intensity profiles for GCF EBT3. Reading is at 488 nm (left), 532 nm (centre) and 670 nm (right). In all cases: irradiation at 405 nm with 1.5 mJ per bleaching point. Scale bar is 40 μm.

The method based on the application of the Rayleigh’s criterion on a two-point irradiation with the 405 nm CW source is graphically shown in Fig. 6. From the analysis, we determined a resolution equal to $(18.3 \pm 1.1)~\mathrm {\upmu m}$, $(25 \pm 7)~\mathrm {\upmu m}$, and $(18.1 \pm 1.1)~\mathrm {\upmu m}$ for 488, 532 and 670 nm, respectively. If we compare these results with previous measurements (Fig. 5) at 1.5 mJ energy we have: $\hbox {FWHM}_\text{488}\sim 20 \upmu m$, $\hbox {FWHM}_\text{532}\sim 22 \upmu m$, $\hbox {FWHM}_\text{670}\sim 13 \upmu m$, here defined as the average FWHM value between the minor and major axes. Surprisingly, the resolution at 670 nm appears higher than 532 nm, comparable to that of 488 nm. This evidence, that seems to be counter-intuitive, may be rationalised by the fact that the maximum of absorption of the active layer after irradiation lies in the green region of the visible spectrum (Supplementary Figure 7).

### Resolution for 365 nm irradiation

**Fig. 7:**
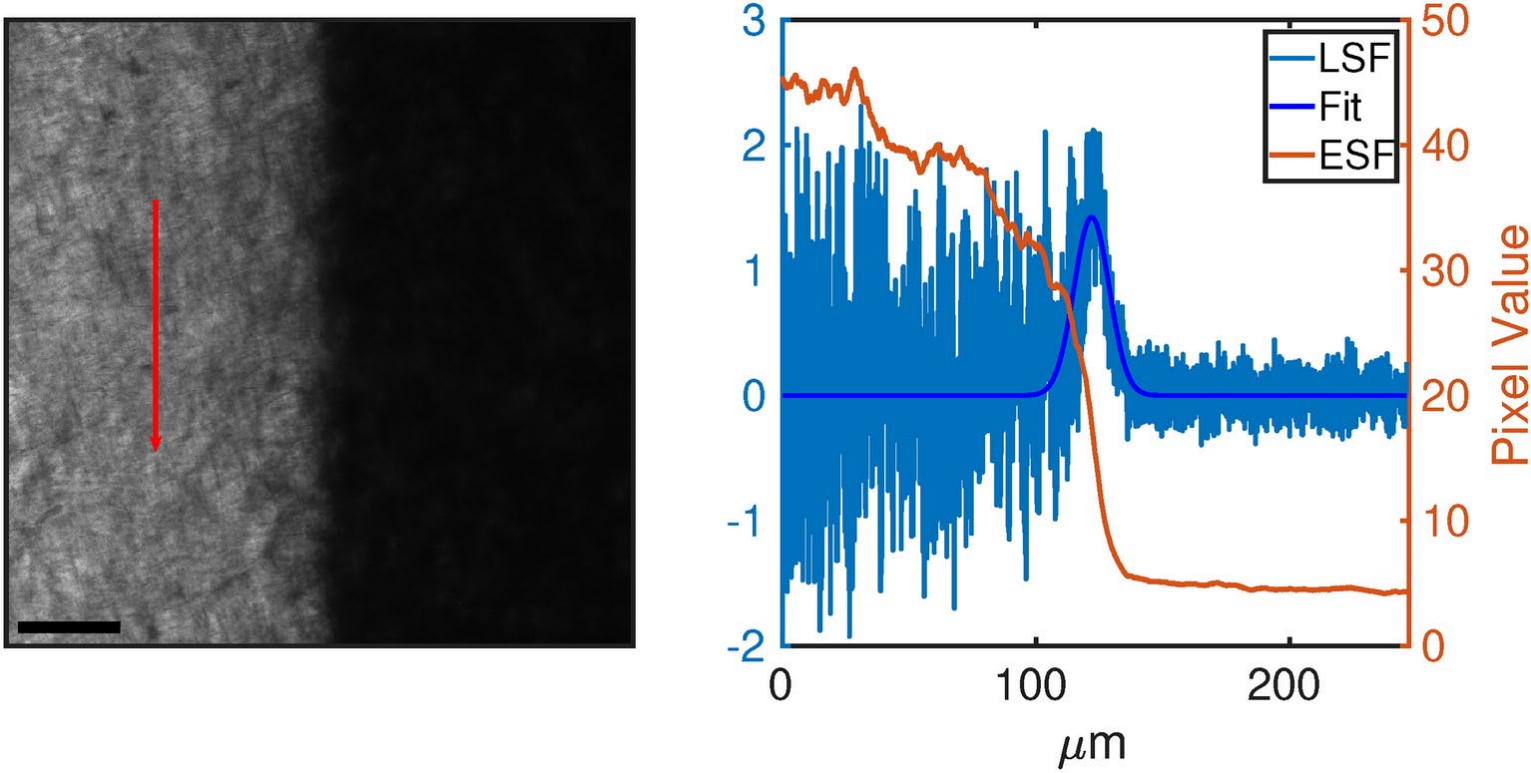
Left: edge profile acquired through the confocal microscope setup. Right: the orange curve is the ESF mediated over the vertical direction of the edge region image. Blue curves represent the LSF, i. e. the gradient of the ESF, and its Gaussian fit. Scale bar is 40 μm.

**Fig. 8:**
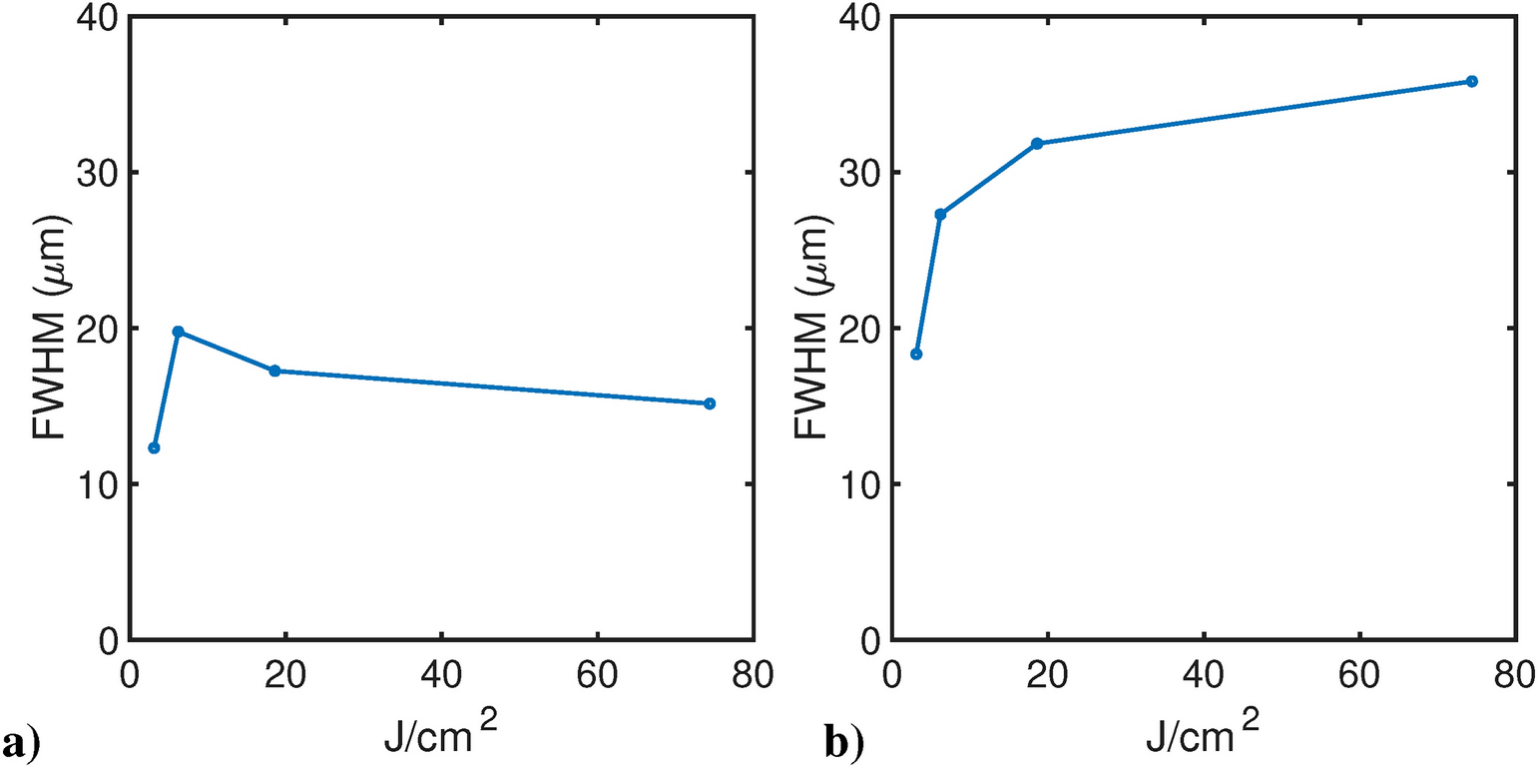
Average spatial resolution for 365 nm irradiation in the case of GCF EBT2 (**a**) and GCF EBT3 (**b**) at four different radiant exposures: 3.1 J/cm^2^, 6.2 J/cm^2^, 18.6 J/cm^2^ and 74.4 J/cm^2^ obtained after an irradiation of, respectively, 5 min, 10 min, 30 min and 4 hours with 365 nm at 10.33 mW/cm^2^.

Regarding the measurements of the GCF resolution for 365 nm-induced darkening, Fig. 7a shows the edge profile acquired after 74 J/cm^2^ of 365 nm exposure, imaged on the GCF EBT2. From Fig. 7b it is evident that the darkened region is rather uniform while the region covered by the chart (non-irradiated) is more irregular, thus introducing a strong noise in the derivative. Light diffusion likely produces a drift of the higher plateau of the ESF (Fig. 7, orange line) that, in the derivative process, adds an offset to the corresponding side of the ESF fit curve (Fig. 7, blue line). This is why a fitting function made of two Gaussian functions bound in their maximum was considered, including two different offset values as represented by Eq. (2) reported in Materials and Methods. Figure 8 reports the resolution averaged along the two axis for both GCF EBT2 (a) and EBT3 (b). The choice to show the average value is due to the impossibility to perfectly align the USAF-1951 target with the films’ weft. These data confirm the trend observed with 365 nm irradiation and correspond to an increase of average FWHM at increasing irradiance.

As is always the case, the measured resolution should be considered as the result of a convolution process between the “intrinsic” response of the film and the optical method (confocal setup, laser or LED irradiation source) for both film writing and reading. The former is influenced by the following factors: (i) the polymerisation process^2,33^, which is characterised by chain propagation, likely affecting the film response to a point-like irradiation by distorting the shape and dimensions of the darkened spot; (ii) light diffusion by the film layers (including the sensitive one), contributing to the same distortion effects; (iii) the filtering effect of the Mylar protective layer. On the other side, the use of a confocal setup (when applicable) allowed for a thorough investigation of the film response to a virtually point-like excitation source.

Overall, for both models the signal-to-noise ratio (SNR) in the determination of the resolution by line profile fitting, improves with increasing radiant exposure (*i.e.* irradiation time in our case), with a maximum for 488 nm reading wavelength. This could be ascribed to: (i) light diffusion by the film layers, depending on the monomer/polymer density; and (ii) an increasing mean length in polymer chains according to the light exposure, which is associated to film darkening in adjacent yet non-irradiated regions respect to the focused light spot.

### Contrast reproduction

**Fig. 9:**
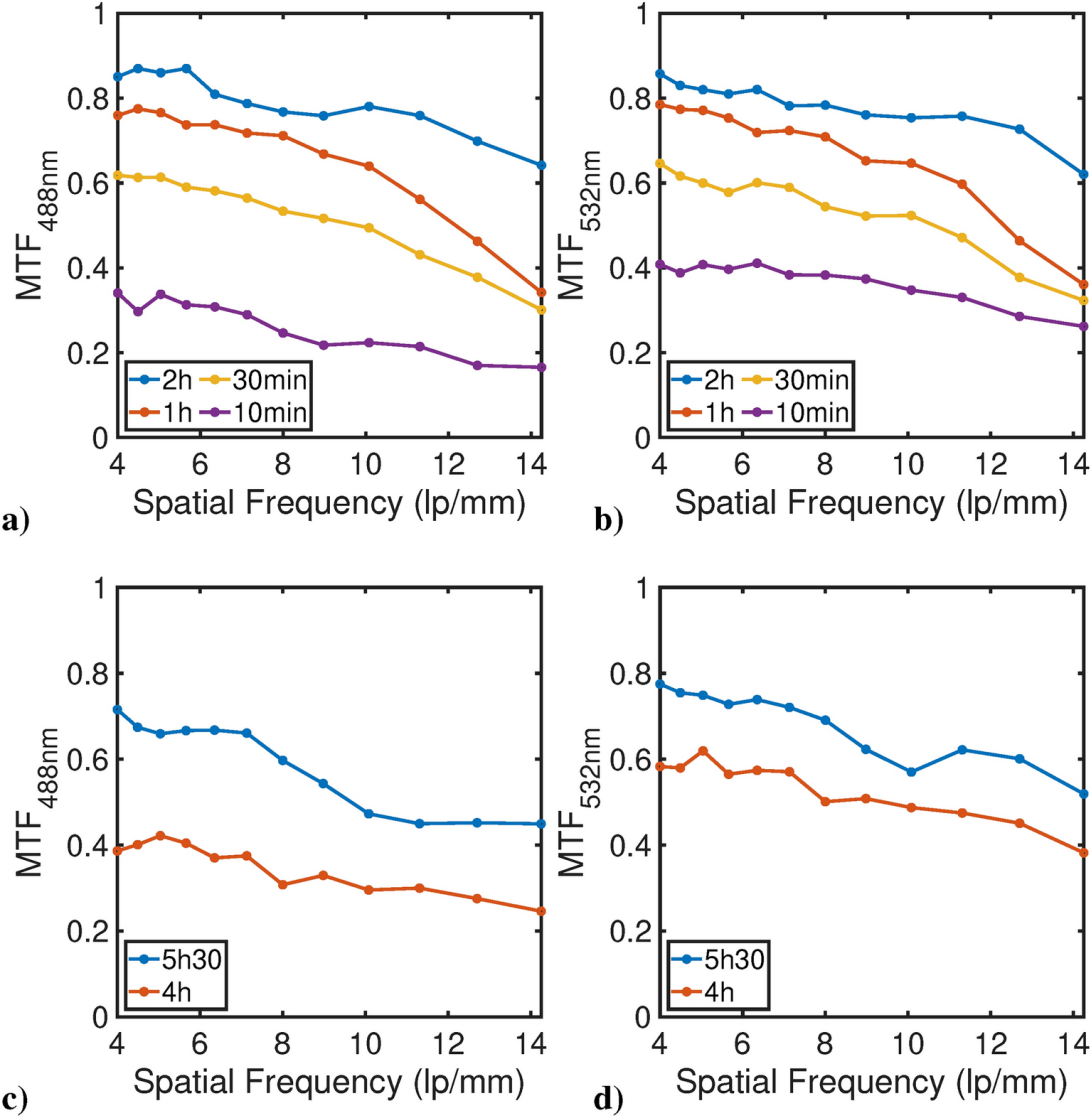
MTF curves from (Eq. 3). 365 nm irradiation at: (**a**) 488 nm reading and (**b**) 532 nm reading. 400 nm irradiation at (**c**) 488 nm reading and (**d**) 532 nm reading.

Figure 9 reports the MTF values corresponding to a radiant exposure ranging between 6 and 36 J/cm^2^ for the 365 nm source and 740–1000 J/cm^2^ for the 400 nm source, respectively. Film reading was performed at 488 nm (a, c) or 532 nm (b, d). As expected, the MTF degrades moving to higher spatial frequencies, while increasing the exposure; at lower radiant exposure, both MTF values and their variations are lower. For the 400 nm irradiation a lower contrast can be noticed, meaning that to produce a film’s darkening comparable with the 365 nm irradiation case, higher irradiation time were necessary. Therefore we can infer that 365 nm is more efficient than 400–405 nm to induce polymerisation. This is reasonable, both recalling the greater photon energy at 365 nm and considering the visible light filtering of the GCF, which is probably more effective in the 400-405 nm region with respect to the UV range.

### Field of View acquisition

**Fig. 10:**
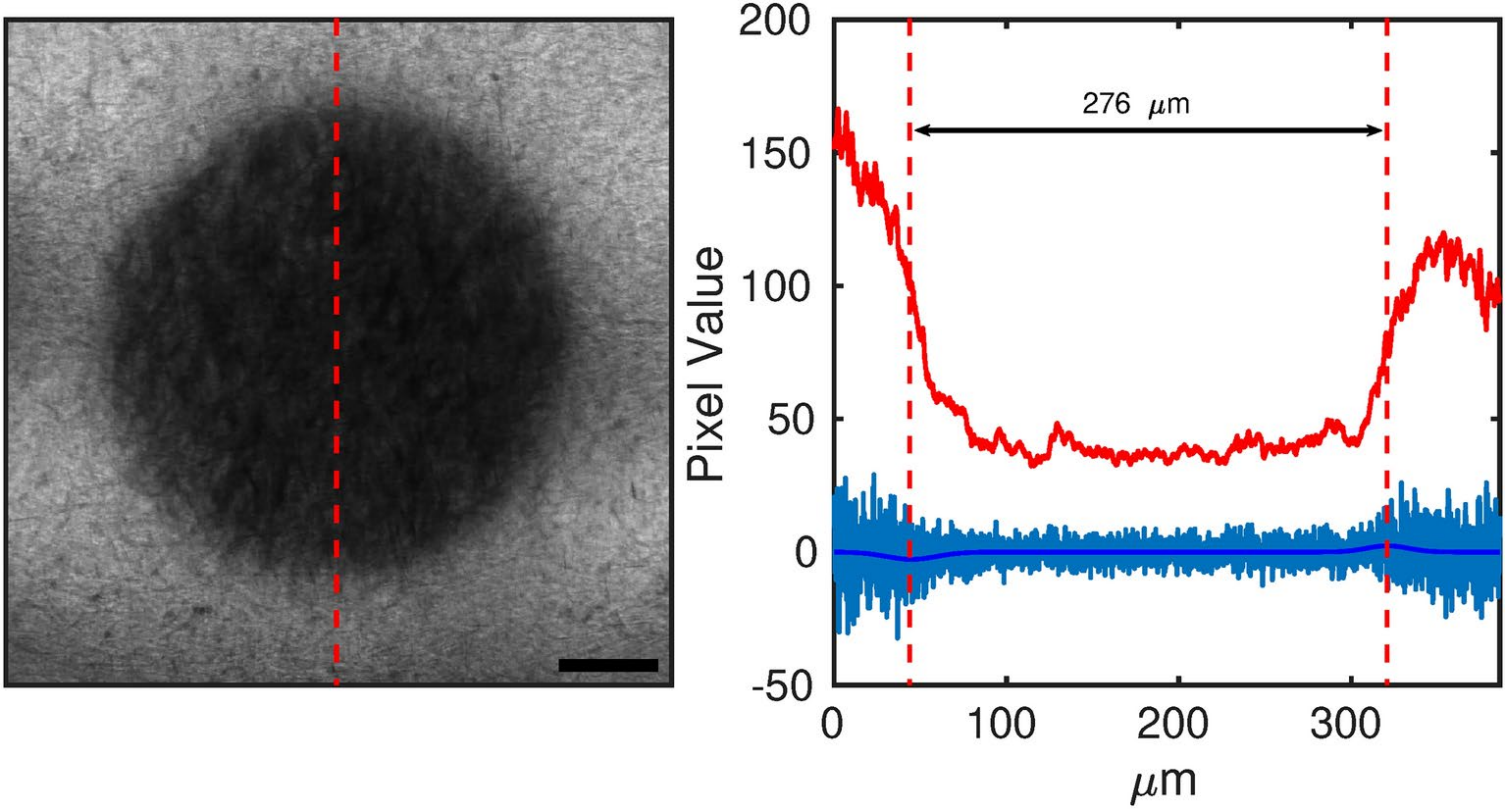
Darkened region (left) obtained by irradiating the EBT3 film with a confocal setup at 360 nm (DAPI line) by a 100 ×/1.4 objective, read with a 40 ×/1.3 objective. Red curve on right is the ESF obtained integrating along red arrow on figure left. Blu curves represent the gradient of the ESF, the LSF, and its Gaussian fit. Gaussian function positions are highlighted by red dashed lines. Scale bar is 60 μm.

Figure 10 shows a direct measurement of a 100× objective (NA 1.4, Leica SP8) supplied with the Leica SP8 confocal setup. The dimension of the FoV is 275 μm. As a comparison the corresponding calculated FoV is 250 μm (considering $FN=25$ and $M_{objective}=100$).

## Conclusions

While originally developed for dosimetry in radiation therapy and equipped with filters to allow use under ambient light conditions, GCF EBT films also exhibit promising potential for applications within the UV-Blue wavelengths. This range holds significant relevance across multiple fields of research. UV light, for instance, is well known for its antimicrobial properties, making it a critical component in the development of new light sources in biomedicine, in addition to being a key element of natural sunlight and an extensively studied region in various medical disciplines. Similarly, blue light, typically covering wavelengths between 380–450 nm, is gaining prominence in photomedicine and regenerative medicine, while also serving as an important excitation source in all optical microscopy techniques.

In our study, we identified the lateral resolution of EBT2 and EBT3 films within the range of 10–30 μm, revealing a dependency on both the irradiation and reading wavelengths, with the latter exerting a more pronounced effect. Notably, the EBT3 model exhibited poorer resolution (*i.e.*, higher FWHM) compared to EBT2 (Figs. 6, 7, 9).

Moreover, we demonstrated the capacity of these films to accurately reproduce contrast across spatial frequencies in the range 4–14 lines/mm, through modulation transfer function (MTF) analysis, with irradiation at wavelengths of 365 nm and 400 nm. To further illustrate their practical applications, we used GCF films to estimate the field of view (FoV) with a high numerical aperture objective (NA = 1.4). By directly evaluating the darkened region, we obtained FoV dimensions with a reasonable deviation (10%) from theoretical predictions.

In summary, GAFchromic films present a highly versatile tool for use as two-dimensional sensors in a wide array of applications, starting with small field imaging in optical microscopy. Notably, in a GCF-confocal setup, the lateral spatial resolution is not diffraction-limited but instead constrained by light diffusion and film polymerisation processes. Nonetheless, GCFs offer a direct and precise means to measure the shape, dimensions, and intensity profile of illumination spots in applications involving cells, tissues, or material samples. These films also offer potential for micro-beam profiling, light dosimetry, and beam profiling for both coherent (laser) and non-coherent light sources.

Future research may explore additional applications of GCF films, including spatial resolution measurements following exposure to ionising radiation-particularly X-ray micro-beams-as well as investigations into axial resolution.

## Supplementary Information

Supplementary Information.

## Data Availability

The authors declare that the data supporting the findings of this study are available within the paper and its supplementary information file. Data sets generated during the current study are available from the corresponding author on reasonable request.
