# Supplementary material for: GAFchromic EBT film lateral resolution and contrast reproduction in the UV-blue range
Source: Sci Rep. 2024 Nov 22;14:28989. doi: 10.1038/s41598-024-78100-3 (PMC11584655; doi:10.1038/s41598-024-78100-3)
Supplement: Supplementary file 1 — Supplementary Information. [file 41598_2024_78100_MOESM1_ESM.pdf]

GAFchromic™ EBT Film Lateral Resolution and Contrast Reproduction in the UV-Blue Range

Francesco Garzella[1,2,+], Giacomo Insero[1,+,*], Antonella Battisti[3], Antonella Sgarbossa[3], Tommaso Mello[1], Franco Fusi[1], and Giovanni Romano[1]

[1]Department of Experimental and Clinical Biomedical Sciences “Mario Serio”, University of Florence, Florence, Italy
[2]Department of Surgical, Medical and Molecular Pathology, and Critical Care Medicine, University of Pisa, Pisa, Italy
[3]NEST, National Research Council - Nanoscience Institute (CNR-NANO) and Scuola Normale Superiore (SNS), Pisa, Italy
*giacomo.insero@unifi.it
+these authors contributed equally to this work

ABSTRACT

The sensitivity of radiochromic films to UV-blue light is increasingly considered for light dosimetry purposes, owing to their bidimensional detection capabilities and ease of use. While film response to radiation intensity has been widely investigated by commercial scanners, spatial resolution studies remain scarce, especially for small field-of-view applications. These are of growing interest due to the antimicrobial or photo-bio-stimulating effects of UV-blue light sources in *in vitro*, *ex vivo* and *in vivo* models, where precise knowledge of irradiation conditions with adequate spatial resolution is crucial. In this study, we report the spatial lateral resolution and contrast reproduction of GAFchromic™ EBT2 and EBT3 models. Upon film irradiation by a 405 nm laser source or 365 nm LED, a confocal microscope setup was employed to read the film response at 405, 470, 488, 532 and 570 nm wavelengths, with radiant exposure of 10—70 J/cm^2. The measured lateral resolution ranged from 8 to 33 μm. The film capability to reproduce contrast across various spatial frequencies (4—14 lines/mm) was evaluated using modulation transfer function analysis with irradiation performed at 365 nm and 400 nm, revealing a pronounced dependency on both radiant exposure and reading wavelength. These results confirm the film capacity to detect and resolve light intensity variability with a $\sim 10\mu$m resolution, with notable applications in micro-beam profiling and light dosimetry.

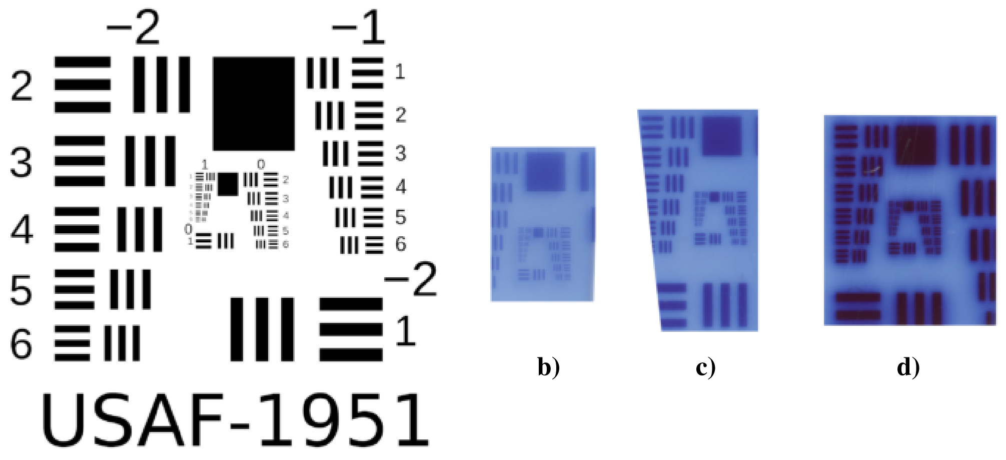

a)

Figure 1. This figure shows the vector map of a USAF-1951 (a), with the group going from -2 to 1, following the definition. Some pattern reproduced on GFC EBT2 at different irradiation time with 365 nm are shown with increasing irradiation time: 5 min (b), 10 min (c) and 1h (d). On (d) it is evident the effect of the diffusion of polymerisation on the pattern that looks surrounded by an halo. This images are acquired using an EPSON Perfection 1640SU scanner with a resolution of 4800 dpi adapted to 1200 dpi.

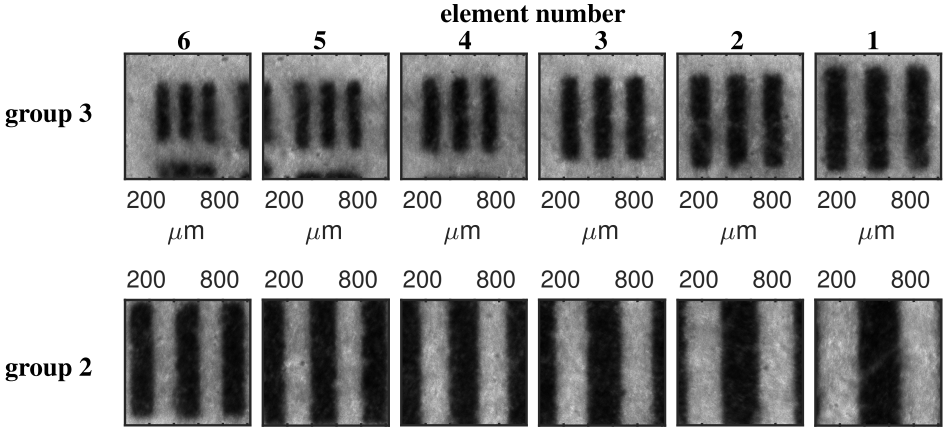

Figure 2. Appearance of GFC EBT3 after irradiation through USAF-1951 for 1h with 365 nm. Top row are the elements of the group 3, while bottom row are the elements of group 2. From left to right the element number decreases from 6 to 1. The spatial frequency decreases following Table 3 from left to right and from top to bottom. Reading was performed at 488 nm.

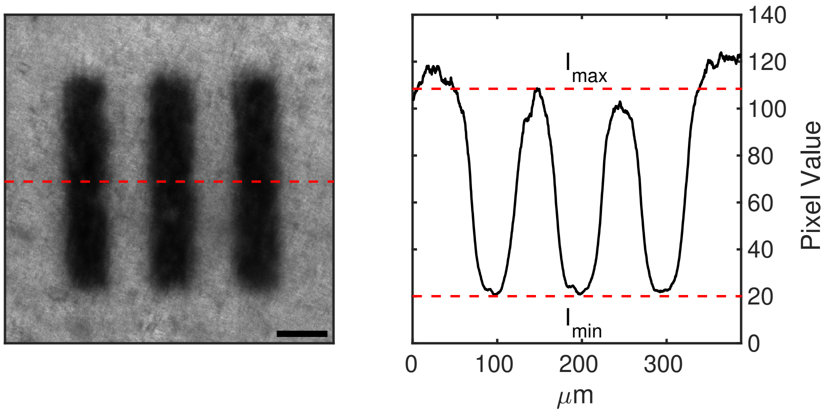

Figure 3. Element 4 of group 6 after irradiation of 1h with 365 nm (left). Profile along the dashed line is reported (right) and used to define I_{max} and I_{min} used to obtained calculated MTF in Figure 6 through Equations 3 and 4. Reading was at 488 nm. Scale bar is 40 um.

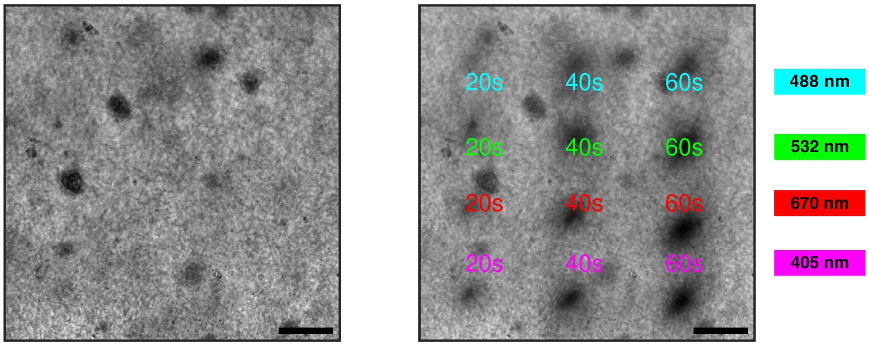

Figure 4. This figure shows GFC EBT3 darkening at different wavelengths (488 nm, 532 nm, 670 nm or 405 nm) and at different irradiation times, after removing the protective layer. The naked active layer can be written by all the wavelengths demonstrating the protective effect of the Mylar layer. Scale-bar is 40 um.

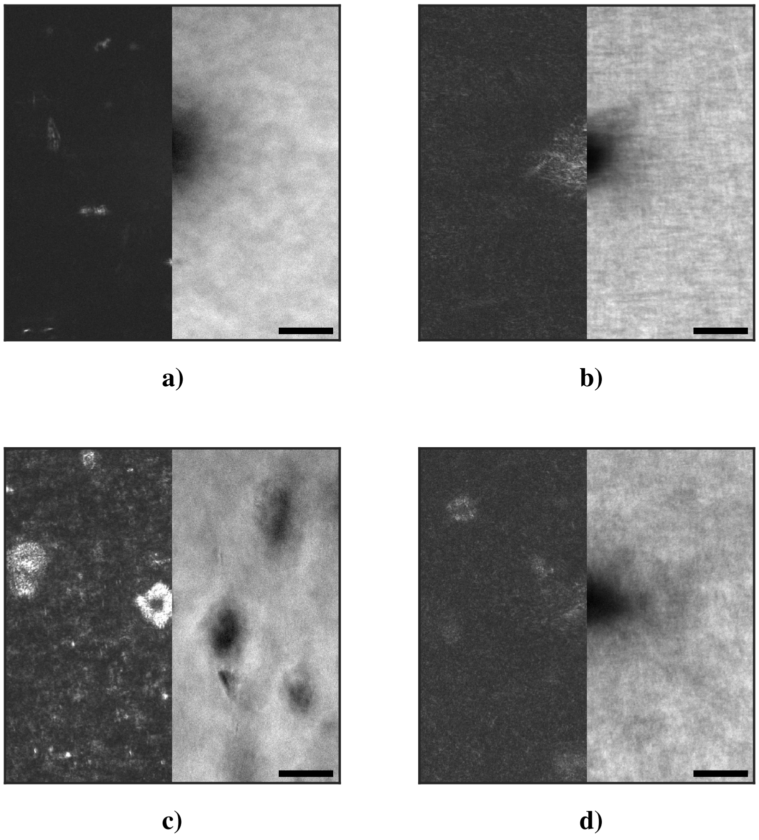

Figure 5. Comparison of the appearance of the active layer (b and d) and the protective layer (a and c) of both GFC EBT2 (a and b) and EBT3 (c and d). All the layers are represented as a comparison of reflection (darker side of all images) and transmission (light side of all images). In both films the active layer (b and d) shows a structured weft due to fabrication process which for EBT2 is more evident (b). The protective layer of EBT3 (c) shows the presence of strongly reflecting structures that are absent in EBT2 protective layer (a). Scale-bar is 15 μm.

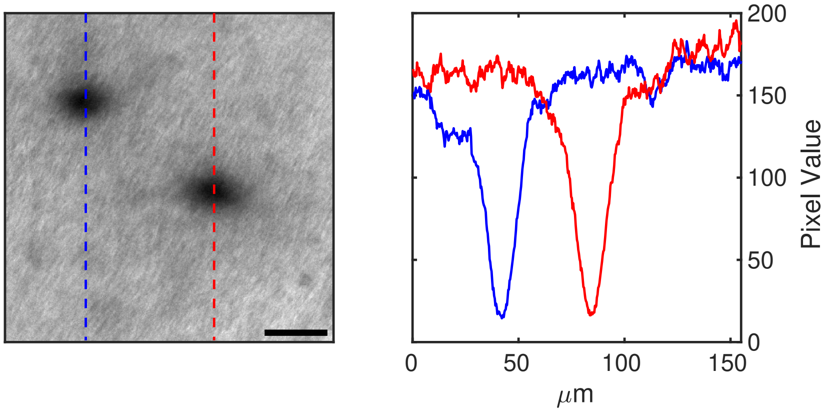

Figure 6. This figure shows that the effect of a stepped writing is comparable with continuous writing. Darkened spot under blue dashed line (left) is obtained after a reading/writing approaches and used to determine the pulsed-laser FWHM in Figure 4. Each point is obtained writing in bleach point mode using 405 nm 40Hz-pulsed laser and read using 488 nm. This spot represents the final appearance after 16 minutes of writing. The darkened spot under red dashed line (left) is obtained after constant writing at 405 nm for 16 minutes. Right: corresponding profiles along the dashed lines. No substantial differences among the two point are evident. Scale-bar is 40 um.

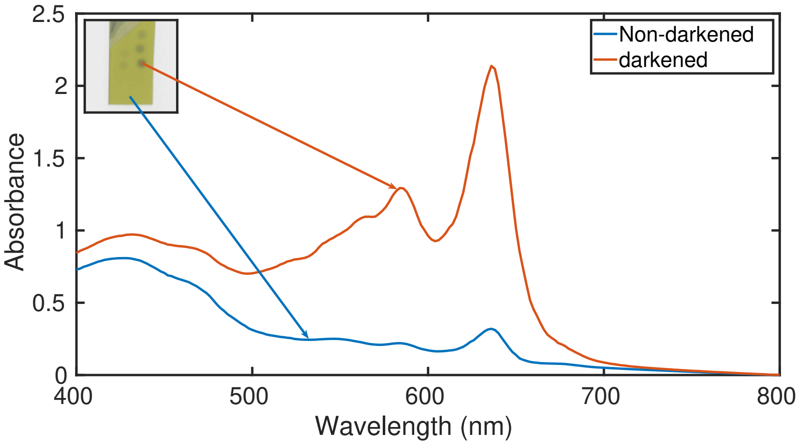

Figure 7. Transmission spectrum of GAFchromic™ EBT3 beofre (solid blue line) and after (solid red line) irradiation with 365 nm fiber LED for 10 minutes. The inset shows the appearance of the film after darkening with spot in different positions for different times. It is evident the change in terms of optical properties, absorbance and colour.

Notes

The function $g(x)$ used to fit the intensity profile reported on Figure 7 consists of two Gauss peaks heaving the same width w overlapped to a linear background, as reported in the following equations:

$$g(x) = a + b \cdot x + c_1 \cdot \exp\left[-\left(\frac{x - x_1}{w}\right)^2\right] + c_2 \cdot \exp\left[-\left(\frac{x - x_2}{w}\right)^2\right] = a + b \cdot x + g_p(x) \quad (1)$$

where a and b represent the coefficients of the linear baseline, c_1 and c_2 the amplitudes of the two Gauss peaks while x_1 and x_2 their positions. We have also denoted with $g_p(x)$ the component that includes only the two Gaussian peaks, distinct from the linear background. The parameter R_d is defined as:

$$R_d = \frac{g_p((x1 + x2)/2)}{\min(c_1, c_2)} \quad (2)$$

while the subscript $d = |x_1 - x_2|$ represent the distance between the two Gauss peak positions. As Rayleigh criterion condition, we defined resolution equal to the distance between the two peaks position $\hat{d}$ when $R \equiv 2$. To properly derive $\hat{d}$, we plot the R_d parameter calculated by using Eq. 2 for the different d values relative to each pair of bleaching points reported in Figure 7. Then we repeat the procedure for the three different reading wavelengths (488, 532 and 670 nm). For each reading wavelength, we fitted the R_d vs d plot with the following exponential function

$$r(d) = y_0 + A \cdot \exp\left(-\frac{d - d_0}{\tau}\right) \tag{3}$$

as shown in red in the following figure, relative to the 670 nm reading wavelength case. The $\hat{d}$ value is obtained in correspondence to the point where $R_d \equiv 2$.

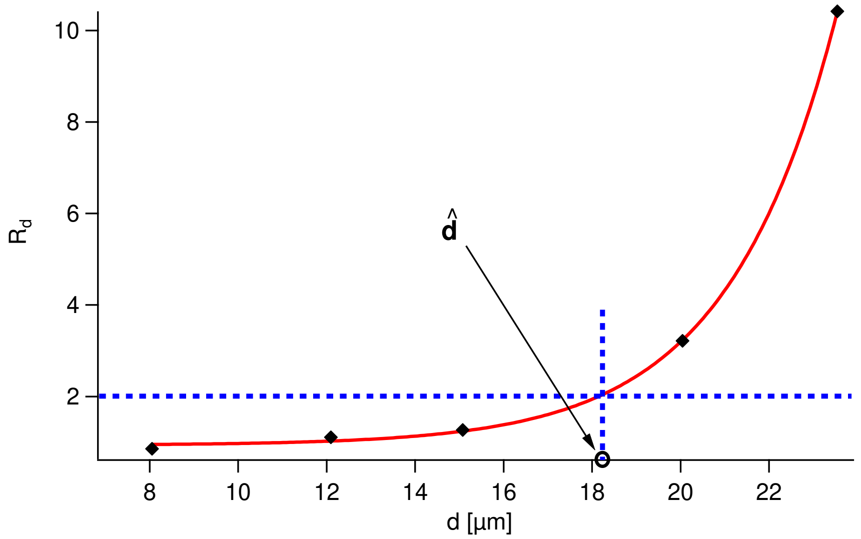

By inverting Eq. 3 and imposing $R_d \equiv 2$ we obtain $\hat{d}$ as follow:

$$\hat{d} = d_0 - \tau \cdot \ln \frac{2 - y_0}{A} \tag{4}$$

with an associated uncertainty of

$$\Delta\hat{d} = \Delta d_0 + \Delta\tau \cdot \left|\ln \frac{2 - y_0}{A}\right| + \Delta A \cdot \left|\frac{\tau}{A}\right| + \Delta d_0 \cdot \left|\frac{\tau}{2 - d_0}\right| \tag{5}$$

where the uncertainty Δd_0, $\Delta\tau$, ΔA, and Δd_0 are obtained from the fitting routine.
